# Investigating Students’ Pre-University Admission Requirements and Their Correlation with Academic Performance for Medical Students: An Educational Data Mining Approach

**DOI:** 10.3390/brainsci13030456

**Published:** 2023-03-08

**Authors:** Ayman Qahmash, Naim Ahmad, Abdulmohsen Algarni

**Affiliations:** 1 Department of Information Systems, King Khalid University, Alfara, Abha 61421, Saudi Arabia; a.qahmash@kku.edu.sa; 2 Department of Computer Science, King Khalid University, Alfara, Abha 61421, Saudi Arabia; a.algarni@kku.edu.sa

**Keywords:** pre-admission criteria, medical education, educational data mining, classification, student performance, Saudi public university

## Abstract

Medical education is one of the most sought-after disciplines for its prestigious and noble status. Institutions endeavor to identify admissions criteria to register bright students who can handle the complexity of medical training and become competent clinicians. This study aims to apply statistical and educational data mining approaches to study the relationship between pre-admission criteria and student performance in medical programs at a public university in Saudi Arabia. The present study is a retrospective cohort study conducted at the College of Computer Science, King Khalid University, Abha, Kingdom of Saudi Arabia between February and November 2022. The current pre-admission criterion is the admission score taken as the weighted average of high school percentage (HSP), general aptitude test (GAT) and standard achievement admission test (SAAT), with respective weights of 0.3, 0.3 and 0.4. Regression and optimization techniques have been applied to identify weightages that better fit the data. Five classification techniques—Decision Tree, Neural Network, Random Forest, Naïve Bayes and K-Nearest Neighbors—are employed to develop models to predict student performance. The regression and optimization analyses show that optimized weights of HSP, GAT and SAAT are 0.3, 0.2 and 0.5, respectively. The results depict that the performance of the models improves with admission scores based on optimized weightages. Further, the Neural Network and Naïve Bayes techniques outperform other techniques. Firstly, this study proposes to revise the weights of HSP, GAT and SAAT to 0.3, 0.2 and 0.5, respectively. Secondly, as the evaluation metrics of models remain less than 0.75, this study proposes to identify additional student features for calculating admission scores to select ideal candidates for medical programs.

## 1. Introduction

The medical profession is a pillar for the well-being of any society. Clinicians play a pivotal role in keeping society healthy and safe against diseases and disasters. Therefore, medical institutions strive to attain the best admissions criteria to recruit medical professionals. One study [1] identified eight selection methods—aptitude tests, academic records, personal statements, references, situational judgment tests, personality and emotional intelligence assessments, interviews and multiple mini-interviews, and selection centers—that are used in different combinations. Another study [2] identified the predictive validity of eight criteria used to select medical students: cognitive factors (previous academic ability), non-cognitive factors (personality, learning styles, interviews, references, personal statements), and demographic factors (sex, ethnicity).

At the onset of medical education, the selection criteria for admission to Saudi medical programs are predominantly based on high school performance coupled with written tests and interviews at some institutions [3]. Currently, in Saudi Arabia, admissions to medical programs are mostly based on the prospective student’s performance on high school percentage (HSP), general aptitude test (GAT) and standard achievement admission test (SAAT). Several studies identified the predictive efficacy of these pre-admission criteria on the performance of medical students vis-à-vis grade point average (GPA). One study [4] at King Fahad Medical City used high school grades, GAT, SAAT and an interview to study the relationship with students’ GPA. Using Pearson correlation coefficient and regression analysis, it found that only SAAT had predictive relevance for the GPA. Another study [5] identified that all pre-admission criteria—high school grades, GAT and SAAT—had good predictive relevance, while SAAT was the most important predictor. Additionally, it identified that the English grade in high school had good predictive relevance.

A study [6] based on statistical analysis at Taibah University found that SAAT and HSP individually, and composite percentage calculated as 40% of HSP, 30% of SAAT and 30% of GAT reliably predicted student performance. GAT individually did not have a significant relationship with students’ GPA. Similarly, a study [7] at King Saud University utilized HSP, SAAT and GAT as predictor variables to predict early academic performance. HSP and SAAT had a significant correlation with early academic performance, while GAT did not. An article [8] explores the relationship of HSP, SAAT and GAT with English performance for first-year students at King Saud Bin Abdulaziz University for Health Sciences. It identified that GAT had higher significance than SAAT in predicting English performance, while HSP had no significance.

Moreover, the latest studies done at King Saud University in 2021 used correlation coefficient and regression analysis to analyze the relationship between GPA, HSP, SAAT and GAT. The study found a significant positive association between pre-admission student scores and their academic performance [9]. Similar studies found that GAT and SAAT have the strongest relationship to student performance in medical college in Saudi Arabia [10]. Other studies of Clinical Biochemistry students at King Adulaziz University showed a weak correlation between SAAT and student performance [11]. Other studies have been conducted to find the relationship between different admission criteria, English test results, and pre-clinical year results in Saudi Arabia [12,13,14].

These previous studies employed formal statistical analysis to study the relationships of pre-admission criteria with the academic performance of medical students. More recently, a new discipline has evolved: educational data mining (EDM), a branch of artificial intelligence and machine learning inspired by brain sciences. “Formal statistical inference is assumption driven in the sense that a hypothesis is formed and then tested against the data. Data mining, in contrast, is discovery driven in the sense that the hypothesis is automatically extracted from the data” [15]. Differing from the standard DM methods, EDM deals with the different hierarchical and non-independent nature of educational data [16]. 

EDM is “concerned with developing methods for exploring the unique types of data that come from educational environments. It can be also defined as the application of data mining (DM) techniques to this specific type of dataset that come from educational environments to address important educational questions” [17]. EDM employs tools and algorithms to obtain insights from data in the educational field. EDM develops methods to explore data stemming from educational settings to understand students and the settings where they study [18]. During the past several years, a wide range of EDM approaches have appeared. A significant amount of research has used EDM to cluster students based on exam results [19,20].

One recent study [21] gives an overview of educational data mining and learning analytics, highlighting their essential role in improving 21st century education; analyzes the concepts; goes over the recent literature; and extracts invaluable information according to the results and outcomes of related studies. It also presents a summary of the main findings, draws conclusions, and provides directions for future research. Based on the results, it asserts that educational data mining and learning analytics fields can significantly influence the current educational system. Similarly, another recent study [22] is a literature survey on predicting teachers’ evaluation in higher education. In this literature survey, the authors depict the various techniques used by researchers across the world to evaluate teachers using data collected on the performance of university students. Results of this research may play a pivotal role in improving higher education system if developed further in broad contexts or fields, as it offers unbiased feedback.

Most existing EDM research has applied clustering, classification, association rule mining, and text mining into educational data [23,24,25]. The EDM community uses four major approaches: prediction models, structure discovery, relationship mining and discovery with models [26]. Educational data mining is capable of producing useful data-driven applications (e.g., early warning systems in schools or the prediction of students’ academic achievement) based on predictive models [27]. Researchers at King Khalid University (KKU) have used Orange data mining tool for EDM for detecting patterns and predicting academic performance of students using online courses offered through learning management systems [28]. The basic idea behind the research was to investigate the educational patterns that affect the academic achievement of KKU students. Researchers have also experimented with various algorithms to predict academic performance at KKU [28].

A prediction model infers a single aspect of the data from a combination of other data aspects (predictive variables similar to independent variables for conventional statistical analysis). A fundamental step in applying a predictive data modelling approach is the selection of features that optimally predict the desired outcome. An article [29] presents the performance analysis of different feature-selection algorithms on an educational dataset. In the present study, the features are predefined as the pre-admission criteria. One study [30] used Artificial Neural Networks, Decision Trees, Support Vector Machines and Naïve Bayes classification techniques to identify the predictive performance of pre-admission criteria such as HSP, SAAT and GAT for the Computer Science and Information College.

This study aims to apply the EDM approach to study the relationship of pre-admission criteria with student performance in medical programs at King Khalid University in Saudi Arabia. The university is situated in the Aseer region in the southern part of the country. It currently has 4171 employees, 3588 faculty members, 29 accredited colleges and 120 departments. A total of 54,291 students are enrolled in diploma, bachelor, master and doctoral programs [31]. The university offers medical education in five domains: medicine, dentistry, applied medical sciences, pharmacy and nursing, through eight colleges in Abha, Khamis Mushayt and Muhay. This study focuses on the students of the medical college. The main aim of the study is to apply educational data mining approaches to study the relationship of pre-admission criteria with student performance in medical programs. In essence, the study addresses the following research question: What is the relationship between current admission criteria and student performance in medical programs?

Further, the study has identified the following objectives in order to answer the research question with the help of EDM techniques:

- Assess the efficacy of weightages given to HSP, GAT and SAT using regression and optimization techniques.
- Compare the performance of selected data mining techniques to predict student performance based on existing admission criteria.
- Recommend revised weightages for admission criteria based upon comparative analysis of data mining techniques using existing weightages and optimized weightages to predict student performance.

The remainder of the paper has been organized into following sections: materials and methods, results, discussion, and conclusion.

## 2. Materials and Methods

The present study is a retrospective cohort study conducted at the College of Computer Science, King Khalid University, Abha, Kingdom of Saudi Arabia between February and November 2022. The admission and registration deanship provided anonymized data for the students enrolled in the five academic years 2015–2016 to 2019–2020. The deanship has made these data available on the university portal as open data to be used by other researchers.

As per the research question and objectives of the study, firstly a correlation was identified to establish the relationship of HSP, GAT, SAAT and admission scores with GPA. Thereafter, linear regression and optimization was carried out to identify the optimized weightages of HSP, GAT and SAAT to be used in admission score calculation. Thereafter, different classification techniques of EDM were applied. Two sets of models were established, first on the basis of current admission scores, and second with the optimized admission scores. Finally, on the basis of comparative analysis of two different sets of models, revised weightages are proposed. 

**Figure 1:**
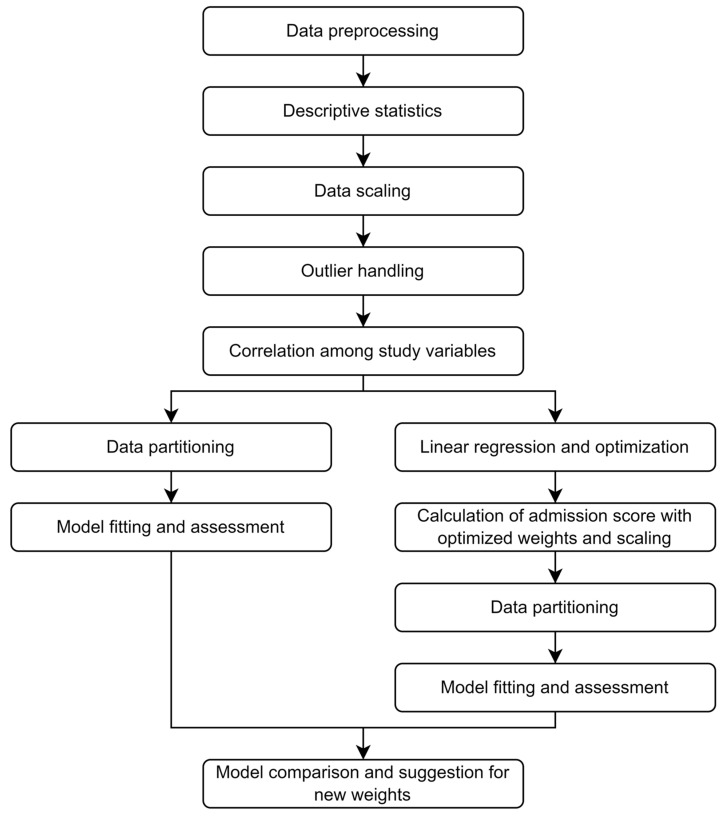
Research Framework.

The following steps were employed to develop and compare the classification models using different data mining techniques: data pre-processing, data scaling and outlier handling, correlation analysis, linear regression and optimization, data partitioning, model fitting and assessment, and model comparisons; Figure 1. These steps are explained in the following paragraphs.

### 2.1. Data Pre-Processing and Descriptive Statistics

Data pre-processing involves loading data, identifying class distribution and handling missing values. Four variables are loaded: HSP, GAT, SAAT and GPA. As expected, the brightest students are enrolled in the medicine program, so their GPA is high. However, the class imbalance problem in educational datasets could hamper the accuracy of predictive models as many of these models are designed on the assumption that the predicted class is balanced [27]. Therefore, to have an equitable class distribution for the target variable GPA, students were divided into two classes: “excellent,” having a GPA more than 4.25, and “good,” having a GPA less than 4.25 but more than 2. Only two students with a GPA of less than 2 were dropped from the study. There was only one record with missing values (all scores were missing), so it was dropped and will not affect the results [32]. Therefore, 962 participants in this study include students having regular status, GPA more than 2, belonging to the college of medicine and enrolled in the five academic years 2015–2016 to 2019–2020. The descriptive statistics are reported in the results section.

### 2.2. Data Scaling and Outlier Handling

Because the numeric data belonged to different ranges, it was essential to scale the data to a common range. Scaling was done with the help of the *PowerTransformer* function from the *sklearn* library [33]. This function supports Box–Cox transformation [34] and Yeo–Johnson transformation [35]. By using maximum likelihood, the ideal parameter for reducing skewness and stabilizing variance was determined. As the data are positive, the Box–Cox method was chosen with the default settings of zero-mean and unit-variance normalization. Numerous studies point to the improvement of results of models by removing outliers [36,37]. Therefore, outliers were removed using the interquartile range (IQR) method. The IQR method can be applied to data that are not normally distributed [38]. This method first calculates the difference between the 25th and 75th percentile values, known as the IQR. Thereafter, the lower and upper bounds are estimated by subtracting the 1.5 times the IQR from 25th percentile value and adding the 1.5 times the IQR to the 75th percentile value, respectively. Finally, the values beyond the lower and upper bounds are considered outliers. The process identified eight such rows that were dropped, and the record set was reduced to 954. 

### 2.3. Correlation Analysis

The Pearson correlation coefficient was calculated using the Python [39] function *pearsonr* from the *scipy.stats* package. It was calculated between HSP, GAT, SAAT, ASC and admission score optimized ASO, and GPA. The methodology of calculation of ASO is described in the next Section 2.4. It gives two values: the correlation coefficient and the *p*-value. Further, the standard error was calculated using Equation (1). As the sample size (N = 954) is large, Equation (1) may be used to compute the standard error, where r is the correlation coefficient [40].

(1) $$Standard\;error=\left(1-r*r\right)/\sqrt{N}$$

### 2.4. Linear Regression and Optimization, and Admission Score Optimized

Two types of models were developed, one with the current admission criteria and the other with optimized weightages of HSP, GAT and SAAT. Therefore, linear regression and optimization techniques were applied to identify the best weights for each admission criterion. The admission scores were recomputed with the optimized weightages derived in linear regression and optimization process, and named the “admission score optimized” (ASO). More details of the Python functions applied are presented in the results Section 3.3.

### 2.5. Data Partitioning

The feature variable admission score was computed through the weighted average of HSP, GAT and SAAT with the current weightages of 0.3, 0.3 and 0.4, respectively, and named the “admission score current” (ASC). The dataset was then partitioned into two sets of training and testing in a ratio of 80:20, with one feature as ASC and the target variable as GPA. This step was repeated after step 2.4 for ASO-based models. 

### 2.6. Model Fitting and Assessment

Five techniques—Decision Tree, Neural Network, Naïve Bayes, K-Nearest Neighbors [41] and Random Forest [42]—were used to fit the model, with ASC being the feature and GPA the target. Further, another set of models were rebuilt using ASO as a feature and GPA as the target variable. The models were assessed and compared on four performance metrics as mentioned in the next step.

### 2.7. Model Comparisons

Four metrics—accuracy, recall, precision, and the F1 score [25,43]—are used to evaluate the performance of a model. Their values depend on the classification confusion matrix, listing the following outcomes:

- True positive (TP): the number of cases correctly predicted as positive,
- False positive (FP): the number of cases incorrectly predicted as positive,
- True negative (TN): the number of cases correctly predicted as negative and
- False negative (FN): the number of cases incorrectly predicted as negative.

Accuracy—representing the effectiveness of the model—is the percentage of correctly predicted results, as per Equation (2). Recall, or sensitivity, is the percentage of correctly predicted positives to total positives, as per Equation (3). Precision, which accounts for the predictive power of the model, is the percentage of correct positive observations, as per Equation (4). F1 score is the harmonic mean of the recall and precision, a measure that balances recall and precision, as per Equation (5).

(2) $$Accuracy=\left(TP+TN\right)/\left(TP+TN+FP+FN\right)$$

(3) $$Recall=TP/\left(TP+FN\right)$$

(4) $$Precision=TP/\left(TP+FP\right)$$

(5) $$F1\;score=2*\left(Precision*Recall\right)/\left(Precision+Recall\right)$$

These four criteria were used to compare the performance of all five models developed using data mining techniques, namely Decision Tree, Neural Network, Random Forest, Naïve Bayes and K-Nearest Neighbors. Similarly, comparison were made between both sets of models based on ASC and ASO feature variables.

## 3. Results

### 3.1. Descriptive Statistics

Table 1 shows the descriptive statistics for all the variables used for the analyses in this study. The sample size is 962, after the removal of one record that had all missing scores. The high school percentage is very high. The GAT scores are marginally higher than the SAAT scores. Moreover, SAAT scores are more spread out than GAT because the standard deviation is higher. Further, the GPA is also higher. The data were scaled and outliers were removed before further analysis. The outliers were identified using the IQR method. Only eight GAT scores were outside the upper and lower bounds, whereas all other scores were within the upper and lower bounds identified by the IQR method. Therefore, the resulting sample size was reduced to 954.

### 3.2. Correlations

All admission criteria—HSP, GAT and SAAT—show a significant correlation with GPA. Nonetheless, GAT has the lowest correlation coefficient (Table 2). Similarly, the feature variables ASC and ASO show a significant correlation with the target variable GPA (Table 2). The correlation coefficient between admission score and GPA was higher for ASO compared to ASC. As the sample size (N = 954) is large, Equation (5) may be used to compute the standard error [40]. Table 2 also shows the standard errors for the correlations, which are very low.

### 3.3. Linear Regression and Optimization

The feature variable admission score current (ASC) was computed using the weighted average of HSP, GAT and SAAT with the current weightages of 0.3, 0.3 and 0.4, respectively. The scores were also computed with new weights derived from the regression and optimization techniques. The regression and optimization techniques were run in Python [39] using the *LinearRegression* function of the *sklearn* package and minimize routine of the *scipy.optimize* package, respectively. The *LinearRegression* function was used to define the function to be used in minimize routine. This function was set to compute and return the mean squared error (MSE) by fitting admission scores with GPA. The *minimize* function iterated the values of weightages with the boundary condition set between 0.2 and 1.0 for all variables (HSP, GAT and SAAT). This resulted in new weightages of 0.311, 0.200 and 0.489 for HSP, GAT and SAT in sixteen iterations. Furthermore, the value of the MSE decreased from 0.7225 to 0.7020. Therefore, the admission score optimized (ASO) was estimated using the weighted sum of HSP, GAT and SAAT with the weightages of 0.3, 0.2 and 0.5, respectively, rounded to one decimal place for simple implementation. In essence, this proposal translates to a 33.33 percent decrease in the weightage of GAT and a 25 percent increase in the weightage of SAAT from the current values. The value of HSP remains unchanged.

### 3.4. Model Fitting 

**Figure 2:**
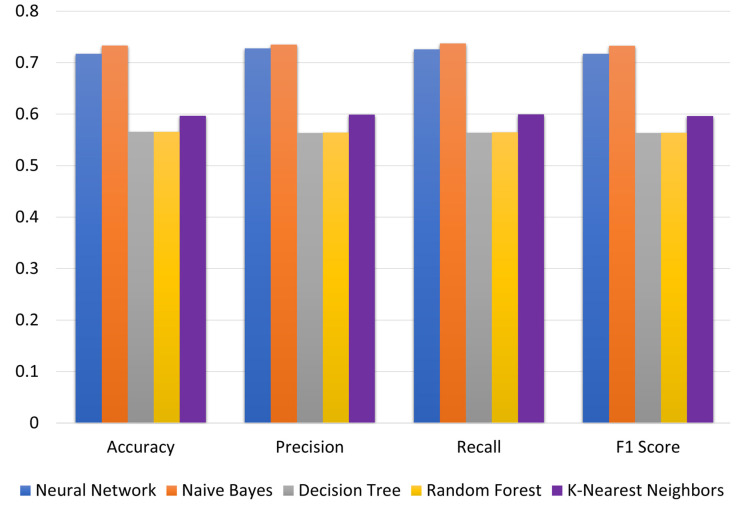
Model comparison for different techniques based on current admission scores (ASC).

Decision Tree, Neural Network, Random Forest, Naïve Bayes and K-Nearest Neighbors were run using ASC as the feature variable and GPA as the target variable. Model evaluation metrics such as accuracy, recall, precision, and the F1 score were estimated using Python’s metrics class from the sklearn package. The comparative charts in Figure 2 were prepared to visualize the differences among different techniques. Naïve Bayes achieves the best performance on all evaluation metrics followed by Neural Network and K-Nearest Neighbors. Decision Tree and Random Forest show the poorest (and similar) performance on all evaluation metrics. The highest values of accuracy, precision, recall and F1 score—0.733, 0.735, 0.737 and 0.737, respectively—are achieved for the Naïve Bayes technique and are within an acceptable range. 

**Figure 3:**
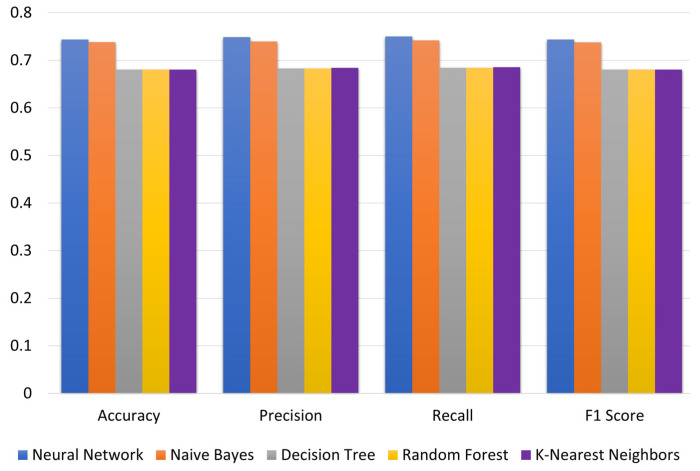
Model comparison for different techniques based on optimized admission scores (ASO).

The models were run using ASO as the feature variable and GPA as the target variable. Figure 3 shows the evaluation metrics. Further, the results show that Neural Network achieves the best performance on all evaluation metrics followed closely by Naïve Bayes. K-Nearest Neighbors, Decision Tree and Random Forest achieve the poorest performance on all evaluation metrics. The highest values of accuracy, precision, recall and F1 score of 0.743, 0.749, 0.750 and 0.743, respectively, are achieved for the Neural Network technique. 

### 3.5. Model Comparisons 

**Figure 4:**
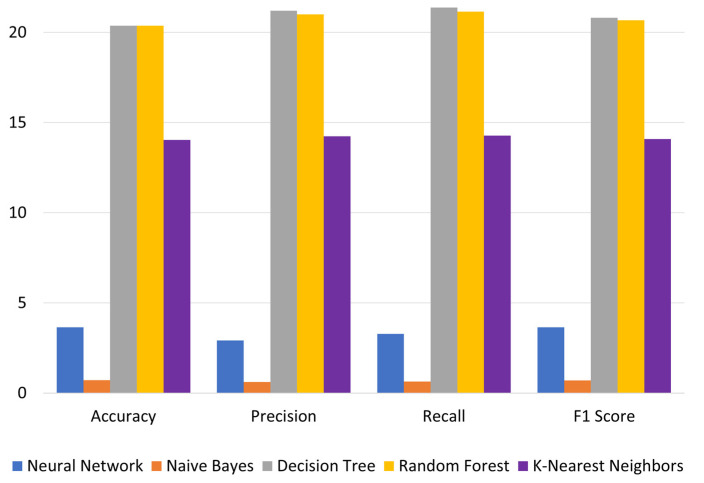
Percentage improvements in evaluation metrics for ASO models over ASC models.

Further, Table 3 demonstrates the differences in the outcomes of models using ASC and ASO values. The performance of all metrics for all techniques improves for ASO models over ASC models. The results show that the evaluation metrics for all techniques for ASO models are above 0.68. Figure 4 depicts the percentage performance gain for all techniques for ASO models over ASC models. The highest (and similar) improvements are achieved for Decision Tree and Random Forest, followed by K-Nearest Neighbors. A slight improvement was achieved for Neural Network and the lowest improvement for Naïve Bayes. The performance of Neural Network and Naïve Bayes was similar. The performance of Decision Tree, Random Forest and K-Nearest Neighbors was similar.

## 4. Discussion

Education is one of the cardinal pillars of any nation that unassailably aids in the acceleration of the nation’s economic development. The recent advancement of technology, especially information technology, in education systems has resulted in an enormous amount of data (“Big Data”) that could be exploited to understand student behavior and progress in acquiring relevant knowledge, as well as their possible future contribution to society. Data can also be used to evaluate the effectiveness of a teacher in molding students’ knowledge-acquiring behavior. Similarly, students’ pre-admission criteria may also be correlated to their educational performance to evaluate the effectiveness of the criteria. 

This study first confirms the significant correlation of all variables—HSP, GAT, SAAT, ASC and ASO—with GPA, as the *p*-values are less than 0.05 and the standard errors are below 0.03. Therefore, all the pre-admission criteria such as HSP, GAT and SAAT may continue to be important factors for calculating admission scores. However, the results of linear regression and optimization techniques show that the weightages allocated to HSP, GAT and SAT are not optimal. The MSE was used to identify optimal weightages. It reached the lowest value of 0.7020 in 16 iterations while keeping the lower boundary values of these variables at 0.2. This resulted in optimized weightages of 0.311, 0.200 and 0.489 for HSP, GAT and SAT, respectively. Finally, the new proposed weightages are 0.3, 0.2 and 0.5 for HSP, GAT and SAT, respectively, rounded to one decimal place for simple implementation.

Furthermore, the models fitted with optimized weightages derived through linear regression and optimization techniques outperform the models fitted with current weightages for each technique. Therefore, this study proposes to revise the weightages for HSP, GAT and SAAT to 0.3, 0.2 and 0.5, respectively. The proposal to reduce the weightage of GAT by 0.1 and increase that of SAAT by 0.1 is consistent with the results of previous studies [4,5,6,7] which identified no significant relationship with the performance of students vis-à-vis GPA. However, SAAT had a significant relationship with GPA in those studies. In one university, an increase in SAAT weightage has already been implemented [8].

Second, for the comparative assessments, Naïve Bayes performs best for current weightages, followed by Neural Network and K-Nearest Neighbors. Decision Tree and Random Forest yield similar performance and produce the lowest evaluation metrics. Neural Network performs best for optimized weightages, followed closely by Naïve Bayes. K-Nearest Neighbors, Decision Tree and Random Forest yield similar performance and produce lower values of evaluation metrics. The outperformance of Neural Networks is well-established in the literature [44,45]. 

The evaluation metrics for the best-performing technique, Neural Networks, reached acceptable levels for accuracy (effectiveness), precision (predictive power), recall (sensitivity) and F1 Score (the balance between the recall and the precision), with values of 0.743, 0.749, 0.750 and 0.743, respectively. Nonetheless, there is room to improve the performance of classification through identifying additional constituents for admission scores in addition to HSP, GAT and SAAT. This study advises identifying and incorporating additional aspects of prospective students for computing admission scores to improve the relationship with GPA and improve the metrics of models.

## 5. Conclusions

This study applied educational data mining techniques, a subfield of artificial intelligence and machine learning inspired from brain sciences, to assess the efficacy of pre-admission criteria to predict the performance of medical students vis-à-vis GPA. The existing features HSP, GPA and SAAT show significant correlation with GPA. However, the study proposes to revise the weightages of HSP, GAT and SAT to 0.3, 0.2 and 0.5, respectively, estimated through the regression and optimization technique in calculating the admission score. These optimized weightages improve the performance of different EDM models compared to the existing weightages of HSP, GAT and SAAT of 0.3, 0.3 and 0.4. In essence, this proposal translates to a 33.33 percent decrease in the weightage of GAT and a 25 percent increase in the weightage of SAAT from the current values, while the value of HSP remains unchanged. Further, the study identifies that Neural Network and Naïve Bayes techniques produce similar results and outperform other techniques. Because the metrics of the models remain less than 0.75, the university may identify and incorporate additional student features to improve the prediction metrics of the models. This research has limited the features to only HSP, GAT and SAAT as per the current university policy. Future research may expand the features to include non-cognitive and demographic factors. Additionally, future studies may apply other brain sciences techniques such as dynamic neural network model types to predict or classify student performance. This will help to identify more capable and deserving students for medical programs.

## Figures and Tables

**Table 1:** Descriptive statistics.

Variables	Mean ± SD ^1^
High school percentage (HSP)	99.23 ± 1.07
General aptitude test (GAT)	90.00 ± 4.72
Standard achievement admission test (SAAT)	89.44 ± 6.80
Grade point average (GPA)	4.09 ± 0.72

^1^ Standard Deviation.

**Table 2:** Pearson correlation coefficient and *p*-value for features with the target variable.

Variables	Pearson Correlation Coefficient	*p*-Value	Standard Error
High school percentage (HSP)	0.3779	0.00	0.0278
General aptitude test (GAT)	0.2814	0.00	0.0298
Standard achievement admission test (SAAT)	0.5144	0.00	0.0238
Admission Score Current (ASC)	0.5135	0.00	0.0238
Admission Score Optimized (ASO)	0.5269	0.00	0.0238

**Table 3:** Evaluation metrics for all models using current and optimized admission scores.

Metrics	Accuracy			Precision			Recall			F1 Score		
Techniques	ASC ^1^	ASO ^2^	% imp ^3^	ASC	ASO	% imp	ASC	ASO	% imp	ASC	ASO	% imp
Decision Tree	0.565	0.681	20.4%	0.563	0.683	21.2%	0.564	0.684	21.4%	0.563	0.680	20.8%
Neural Network	0.717	0.743	3.6%	0.728	0.749	2.9%	0.726	0.750	3.3%	0.717	0.743	3.7%
Random Forest	0.565	0.681	20.4%	0.564	0.683	21.0%	0.565	0.684	21.2%	0.564	0.680	20.7%
Naïve Bayes	0.733	0.738	0.7%	0.735	0.740	0.6%	0.737	0.742	0.6%	0.733	0.738	0.7%
K-Nearest Neighbors	0.597	0.681	14.0%	0.599	0.684	14.2%	0.600	0.685	14.3%	0.596	0.680	14.1%

^1^ percentage improvement; ^2^ admission score current; ^3^ admission score optimized.

## Data Availability

The Deanship of Registration at King Khalid University has made the data of this study available on the university portal as open data to be used by other researchers.

## References

[1] Patterson F., Knight A., Dowell J., Nicholson S., Cousans F., Cleland J. (2016). How effective are selection methods in medical education? A systematic review. Med. Educ..

[2] Ferguson E. (2002). Factors associated with success in medical school: Systematic review of the literature. BMJ.

[3] Al-Sulaiman A.A. (2000). Saudi medical education: Challenges in the new millennium. J. Fam. Community Med..

[4] Al-Rukban M.O., Munshi F.M., Abdulghani H.M., Al-Hoqail I. (2010). The ability of the pre-admission criteria to predict performance in a Saudi medical school. Saudi Med. J..

[5] Albishri J.A., Aly S.M., Alnemary Y. (2012). Admission criteria to Saudi medical schools. Which is the best predictor for successful achievement?. Saudi Med. J..

[6] Murshid K.R. (2013). The predictive value of individual admission criteria on academic performance in a Saudi medical college. J. Taibah Univ. Med. Sci..

[7] Alhadlaq A.M., Alshammari O.F., Alsager S.M., Neel K.A.F., Mohamed A.G. (2015). Ability of Admissions Criteria to Predict Early Academic Performance Among Students of Health Science Colleges at King Saud University, Saudi Arabia. J. Dent. Educ..

[8] Althewini A., Alkushi A. (2020). Predictive validity of Saudi admission criteria for freshmen students’ English performance: Experience of king Saud Bin Abdulaziz university for Health sciences. J. Lang. Teach. Res..

[9] Alhurishi S.A., Aljuraiban G.S., Alshaikh F.A., Almutairi M.M., Almutairi K.M. (2021). Predictors of students’ academic achievements in allied health professions at King Saud University: A retrospective cohort study. BMC Med. Educ..

[10] Althewini A., Al Baz N. (2022). Prediction of Admission Tests for Medical Students’ Academic Performance. Adv. Med. Educ. Pract..

[11] Alamoudi A.A., Fallatah H.I., Eldakhakhny B.M., Kamel F.O., AlShawwa L.A., Elsamanoudy A.Z. (2021). Relationship between admission criteria and academic performance in basic science courses in health science colleges in KAU. BMC Med. Educ..

[12] Dabaliz A.-A., Kaadan S., Dabbagh M.M., Barakat A., Shareef M.A., Al-Tannir M., Obeidat A., Mohamed A. (2017). Predictive validity of pre-admission assessments on medical student performance. Int. J. Med. Educ..

[13] Jawhar S.S., Al Makoshi M., Alhawsawi S., Alkushi A. (2021). Validating English Language Entrance Test at a Saudi University for Health Sciences. Arab World Engl. J. Vol..

[14] Al-Qahtani M.F., Alanzi T.M. (2018). Comparisons of the predictive values of admission criteria for academic achievement among undergraduate students of health and non-health science professions: A longitudinal cohort study. Psychol. Res. Behav. Manag..

[15] Romero C., Ventura S. (2007). Educational data mining: A survey from 1995 to 2005. Expert Syst. Appl..

[16] Baker R.S.J.D., Yacef K. (2009). The state of educational data mining in 2009: A review and future visions. J. Educ. Data Min..

[17] Romero C., Ventura S. (2020). Educational data mining and learning analytics: An updated survey. Wiley Interdiscip. Rev. Data Min. Knowl. Discov..

[18] Algarni A. (2016). Data mining in education. Int. J. Adv. Comput. Sci. Appl..

[19] Vandamme J.-P., Meskens N., Superby J.-F. (2007). Predicting academic performance by data mining methods. Educ. Econ..

[20] Bresfelean V.P., Bresfelean M., Ghisoiu N., Comes C.-A. Determining students’ academic failure profile founded on data mining methods. Proceedings of the ITI 2008-30th international conference on information technology interfaces.

[21] Lampropoulos G. (2023). Educational Data Mining and Learning Analytics in the 21st Century. Encyclopedia of Data Science and Machine Learning.

[22] Ordoñez-Avila R., Reyes N.S., Meza J., Ventura S. (2023). Data mining techniques for predicting teacher evaluation in higher education. A SYSTEMATIC literature review. Heliyon.

[23] Mohsin M.F.M., Hibadullah C.F., Norwawi N.M., Abd Wahab M.H. Mining the student programming performance using rough set. Proceedings of the 2010 IEEE International Conference on Intelligent Systems and Knowledge Engineering.

[24] Zaiane O.R. Building a recommender agent for e-learning systems. Proceedings of the International Conference on Computers in Education.

[25] Balaji P., Alelyani S., Qahmash A., Mohana M. (2021). Contributions of Machine Learning Models towards Student Academic Performance Prediction: A Systematic Review. Appl. Sci..

[26] Baker R.S.J.d., Peterson P., Baker E., McGaw B. (2010). Data Mining. International Encyclopedia of Education.

[27] Wongvorachan T., He S., Bulut O. (2023). A Comparison of Undersampling, Oversampling, and SMOTE Methods for Dealing with Imbalanced Classification in Educational Data Mining. Information.

[28] Abdelmagid A.S., Qahmash A.I.M. (2023). Utilizing the Educational Data Mining Techniques" Orange Technology" for Detecting Patterns and Predicting Academic Performance of University Students. Inf. Sci. Lett..

[29] Zaffar M., Hashmani M.A., Savita K.S. Performance analysis of feature selection algorithm for educational data mining. Proceedings of the 2017 IEEE Conference on Big Data and Analytics (ICBDA).

[30] Mengash H.A. (2020). Using data mining techniques to predict student performance to support decision making in university admission systems. IEEE Access.

[31] Statistics. https://www.kku.edu.sa/en/statistics.

[32] Harrell F.E. (2001). Regression Modeling Strategies: With Applications to Linear Models, Logistic Regression, and Survival Analysis.

[33] Pedregosa F., Varoquaux G., Gramfort A., Michel V., Thirion B., Grisel O., Blondel M., Prettenhofer P., Weiss R., Dubourg V. (2011). Scikit-learn: Machine learning in Python. J. Mach. Learn. Res..

[34] Osborne J. (2010). Improving your data transformations: Applying the Box-Cox transformation. Pract. Assess. Res. Eval..

[35] Yeo I.-K., Johnson R.A. (2000). A new family of power transformations to improve normality or symmetry. Biometrika.

[36] Vinutha H.P., Poornima B., Sagar B.M., Satapathy S., Tavares J., Bhateja V., Mohanty J. (2018). Detection of Outliers Using Interquartile Range Technique from Intrusion Dataset. Information and Decision Sciences. Advances in Intelligent Systems and Computing.

[37] Dash C.S.K., Behera A.K., Dehuri S., Ghosh A. (2023). An outliers detection and elimination framework in classification task of data mining. Decis. Anal. J..

[38] Walfish S. (2006). A review of statistical outlier methods. Pharm. Technol..

[39] Van Rossum G., Drake F.L. (2011). The Python Language Reference Manual.

[40] Gnambs T. (2022). A Brief Note on the Standard Error of the Pearson Correlation. https://psyarxiv.com/uts98.

[41] Tan P.-N., Steinbach M., Kumar V. (2016). Introduction to Data Mining.

[42] Biau G., Scornet E. (2016). A random forest guided tour. Test.

[43] Sokolova M., Japkowicz N., Szpakowicz S. Beyond accuracy, F-score and ROC: A family of discriminant measures for performance evaluation. Proceedings of the Australasian Joint Conference on Artificial Intelligence.

[44] Shahiri A.M., Husain W., Rashid N.A. (2015). A review on predicting student’s performance using data mining techniques. Procedia Comput. Sci..

[45] Kaur P., Singh M., Josan G.S. (2015). Classification and prediction based data mining algorithms to predict slow learners in education sector. Procedia Comput. Sci..

